# Hospital Construction

**Published:** 1908-01-04

**Authors:** 


### Hampstead General Hospital.

### SOME RECENT HOSPITAL CONSTRUCTION.

[We shall be glad to have from any reader particulars of other hospital construction or new buildings of importance during the last two years.]

During the year we have taken some pains to bring out clearly what constitutes the essentials of a modern general hospital. In our article published in July 1907, page 433, we brought out clearly that no population is justified in incurring a large expenditure upon hospital buildings unless it is prepared to maintain those buildings in efficiency, and fully occupied, when they are completed and ready to receive patients. Unfortunately the modern tendency in hospital construction has sometimes proved that the desire to extend, and ever to increase the amount of work undertaken by a given hospital, is so strong that financial considerations which ought to have the first place in the minds of prudent men before they engage in the luxury of bricks and mortar, are too often relegated to the background. The public are beginning to realise this fact, and unless more prudence is shown by those responsible for hospital buildings of all kinds, in London especially, they must expect to find that the public will refuse to contribute money for these enterprises; so it may come to pass that a great hospital which has enjoyed many years of public confidence and prosperity may find itself in a most critical financial position.

Two great schemes of hospital reconstruction have been successfully carried out in the metropolis in the last decade. One is the practical rebuilding of the London Hospital, which has entailed an expenditure of £491,703, a full account of which appeared in our issue for August 17, page 537. Of this sum £340,221 has been supplied by the public, mainly through the energy of the Chairman, the Hon. Sydney Holland, *i.e.*, £130,000 by a few donors of large gifts for specific purposes, and the balance of £210,000 by responses to dinners and other forms of appeal.

The second great scheme is that of Guy's Hospital, which has been successfully carried out by Mr. H. Cosmo Bonsor, the Treasurer. In the Guy's Hospital scheme, which represents an expenditure of upwards of £400,000, reduced by keen business methods to about £350,000, we have an example of hospital finance at its best. The interesting story of this great reconstruction of one of our oldest and most famous hospitals is told in an article which appeared in The Hospital of November 16, page 189. The money for this reconstruction has been raised from the public at a cost of under 5 per cent., and the Treasurer has been enabled during the last twelve years, in addition to this fine stroke of finance, to increase the average income of Guy's Hospital by no less a sum than £54,000. We believe there is no parallel in the history of voluntary hospitals for this financial achievement. Never in the history of charity has a splendid work been better done than that which has been accomplished at Guy's Hospital during the treasurership of Mr. H. Cosmo Bonsor.

At the Royal Free Hospital two new operationtheatres with a sterilising room and other offices, and a new mortuary, have been completed and brought into use. They have the merit of being reasonably small, and the arrangements, considering the difficulties which had to be overcome, reflect credit upon the architects, Messrs. W. Harvey and A. T. Snell. We gave a plan and full particulars of this reconstruction in our issue of December 7, page 269.

During the year His Royal Highness the Prince of Wales laid the foundation-stone of the new Glasgow Royal Infirmary, which is being rebuilt from plans upon a scheme prepared by Sir Henry Burdett and Dr. D. J. Mackintosh in conjunction with Mr. James Miller, who are jointly responsible for the plans. We published the plan of the admission block which forms a novel feature in this scheme in our issue of April 13, 1907, page 46. We believe that this block is likely to form a new unit in hospital construction, and that the whole scheme, which contains many novel features, will be found very helpful to those interested in hospital construction, when the whole of the plans are published, as they are to be very shortly.

Through the generosity of Mr. W. F. D. Smith, the removal of King's College Hospital to a site in Camberwell is assured. As a result of a limited competition adjudicated on by Mr. Rowland Plumbe, Mr. W. A. Pite was appointed architect. The decision of the assessor was in every way a right one; Mr. Pite's plans showed a grasp of the subject and a knowledge of detail which were only approached by one other competitor, whose plans were placed second. No hospital building of anything like the importance of this one has been undertaken in London since the erection of St. Thomas's Hospital some forty years ago; and the completion of this great scheme will be looked forward to with the liveliest interest by all engaged in hospital work.

The question as to the rebuilding or removing of St. Bartholomew's Hospital was decided on the advice of a specially appointed Committee in favour of rebuilding on the present site with part of that of Christ's Hospital. The first result of this decision in the shape of a new out-patient department has just (November 1907) been completed and opened to patients. The new building has evidently been thought out with great care; and, while it cannot be said to show extravagance, no expense has been spared to make it complete and up-to-date. With such an immense number of patients to deal with it was clearly impossible to plan the whole of the departments on one floor. The ground floor, indeed, is wholly taken up with the large waiting hall, calculated to seat between 800 and 900 patients, the dispensary, and the casualty department. On the first floor are the medical and surgical rooms, and on the two upper floors are the special departments, ear and throat, eye, skin, dental, electric, etc. Besides the staircase, there are two lifts available for taking weak or crippled patients up and down. A new kitchen for the service of the whole hospital and a series of out-patient beds are also included in the scheme.

At the Seamen's Hospital, Greenwich, some interesting—though not extensive—improvements have been carried out. Following on a recommendation made by the Committee of the King's Fund, who made a special grant for the purpose, the old deal floors in some of the wards have been covered with a plastic material which forms a hard impervious surface entirely free from joints. The opportunity was taken to form large openings between the old small rooms, and so to make comparatively large wards. The result is a great improvement in the appearance and ventilation of the wards and considerable economy in nursing. A new out-patient waiting hall has been built, and the out-patient rooms have been much improved. By shifting the dispensary to an adjoining room it has been possible to arrange a room for the administration of anæsthetics and a sterilising room, both communicating with the operation theatre. The sanitary offices throughout the hospital have been much improved, and a much needed want will, it is hoped, shortly be met by the formation of isolation rooms for cases of infectious disease pending their removal to an isolation hospital.

By the addition of a ward block for general patients, one for paying patients and a small out-patient department, the Hampstead General Hospital is now complete. Started some years ago in one small house, and subsequently enlarged by the addition of two other houses, the institution has grown to its present dimensions, and must be ranked as a full-grown general hospital. The reforms in the medical staff suggested by the Committee of the King's Fund having been strenuously opposed by the medical fraternity of Hampstead, the whole question, including also the amalgamation with the out-patient department of the now defunct North-West London Hospital, was referred to a small committee of subscribers, who drew up a report which was referred to the general body of subscribers, with the result indicated on the preceding page.

An inquiry into the expenditure by the Guardians of Hammersmith on their new infirmary brought out some strange revelations of the sort of scale considered reasonable by those responsible for the outlay. Amongst other things, it appeared that the whole of the wiring for the electric light was run in duplicate—a precaution against possible breakdown, which was not thought necessary at Buckingham Palace.

In the early part of 1907 the Guardians of the Edmonton Union invited plans from a selected number of architects in competition for their new union and infirmary, and as a result the design of Mr. Stewart Hill was accepted. Without seeing the whole of the designs it is difficult to say much in the way of criticism, but the selected plan is certainly most unsatisfactory in some ways. The buildings are very crowded on the site, and the aspect of all the ward blocks, without exception, is east and west, instead of being north and south. On the face of it, there does not appear to be any valid reason for this arrangement. The site is a spacious one, apparently open on all sides, and it certainly would seem that a much superior plan might have been devised. Now that the nursing and the treatment in these Poor-law infirmaries is so very much improved to what it used to be, it seems a most unfortunate thing that a new building like this should be projected, which really is very much behind the times in point of proper hospital planning.

At the Evelina Hospital for Sick Children a much-needed out-patient department has been added, together with additional rooms for the nursing staff. The wretchedly inadequate rooms in which the out-patient work of this excellent hospital was carried on have been replaced by spacious well-lighted rooms, properly equipped for the work, and including a well-appointed operating-theatre. The area of site available was not large enough to plan the consulting rooms all on the ground floor. The surgical department is, therefore, on the first floor, but access is made easy by an electric lift with " push-button " control. Above the nurses' rooms is a flat roof, on to which the children are carried in fine weather. A marble tablet records the fact that the cost of the out-patient department was defrayed by the " Trustees of the Ada Lewis bequest."

The London Fever Hospital in Liverpool Road is now undergoing a partial reconstruction which will, it is hoped, bring it into line with the best Fever hospitals in the country. The committee have had under consideration for years past a complete scheme of re-building, and every addition that has been made was planned to fit in with the general scheme. Recognising the impossibility of carrying out the larger scheme for lack of funds, the committee wisely resolved so to inprove the existing buildings that they would be in every way fitted for the work. The buildings were designed more than 50 years ago, and were then considered far in advance of anything that had gone before. The main wards were double, and had four rows of beds between the opposite walls. One half of each ward has been demolished, and the remaining half converted into an excellent ward. New windows have been put in, greatly increasing both light and ventilation, and new teak floors take the place of the old deal. The ward kitchens and sanitary offices have been entirely reconstructed and brought up-to-date. On each side a second wing two stories high replaces the old one-story wings, and makes up for the loss of beds by the demolition of half the main ward. There remains to be built a block for measles, and for the isolation of doubtful cases and complications, and the work of reconstruction will be complete. The work that is done by this hospital is one that cannot be undertaken by the Metropolitan Asylums Board, and which is of incalculable value to the community.

The re-modelling, or practical re-building of the City of London Lying-in Hospital in the City Road has been completed, and, though the policy of retaining the hospital on so unsuitable a site may be questioned, it must be admitted that the new building is a vast improvement on the old one. The plans are by Messrs H. H. and M. E. Collins, who were placed first in a limited competition.

In the provinces the most important work which has been taken in hand is the re-building of the Royal Infirmary, Manchester. The question of removing from the old site in the centre of the City was, after years of fighting, decided, and a site was acquired in Oxford Street. The site is certainly a very inadequate one, and the buildings cannot fail to be crowded. Mr. E. T. Hall was appointed architect after a limited competition; but the wisdom of the decision is open to grave doubts, and certainly the published plans show one very important error, namely, the double corridor from which the wards are approached. This arrangement, which adds just 100 per cent. of unnecessary length to the corridors, must increase enormously the difficulty of supervising and of administration.

A competition was held at Sunderland in the early part of the year for a large children's hospital, the first place being awarded to Messrs. Armstrong and Wright. The plan is in the form of the letter H, with the administration block in the centre. The wards are largely modelled on those at the Derby Hospital, and do not present any novel feature. The mistake has been made of placing the out-patient department under one of the wards. Whether this arose from want of space or from a mistaken idea of economy we do not know; but it is a most unfortunate error. It is more necessary to isolate completely the out-patient department in a children's hospital than in any other, and this cannot be efficiently done if it is placed in the same building as the wards.

At the Wolverhampton General Hospital plans were invited in competition for a nurses' home, and the successful competitor was Mr. Arthur W. Worrall. There is nothing specially remarkable about the plans, which appear to answer the requirements fairly well.

At Leeds the Public Dispensary having been displaced by a street improvement, the institution has been re-built on another site from plans by Messrs. Bedford and Kitson, of Leeds. The plan was published in The Hospital of August 26, 1905, and is very carefully thought out and well contrived. The lighting of every part is specially commendable.

At Warrington a new Nurses' Home and an additional ward block have been built. These were illustrated and described in The Hospital of November 16, 1907. The architects are Messrs. William and Segar Owen, of Warrington.

A very excellent and well-planned hospital for skin diseases has been erected at Manchester, designed by Messrs. Worthington and Son, who were placed first in a limited competition, Mr. Keith Young being the assessor. Manchester is certainly well-equipped with special hospitals, and there is no skin hospital in London to compare with the one in question.

At Salford extensive re-modelling and enlargement of the Royal Infirmary is to be carried out from plans by Mr. Ely. This also was the result of a limited competition in which the committee were advised by Mr. Keith Young. The problem set to the competitors was a difficult one, but Mr. Ely showed considerable knowledge of the subject, and much skill in dealing with a restricted site.

### Sanatoria.

The Winsley Sanatorium at Limpley Stoke, near Bath, was begun in 1903 and opened in 1905. The plan, which was illustrated in The Hospital of January 28, 1905, is a single one, and the wards, which are for one patient each, all face south. The cost works out at £329 per head for 20 beds—not an extravagant sum for so small a number of beds.

